# Supplementary figures and images for: EGFR controls transcriptional and metabolic rewiring in KRASG12D colorectal cancer
Source: EMBO Mol Med. 2025 May 6;17(6):1355–92. doi: 10.1038/s44321-025-00240-4 (PMC12162862; doi:10.1038/s44321-025-00240-4)

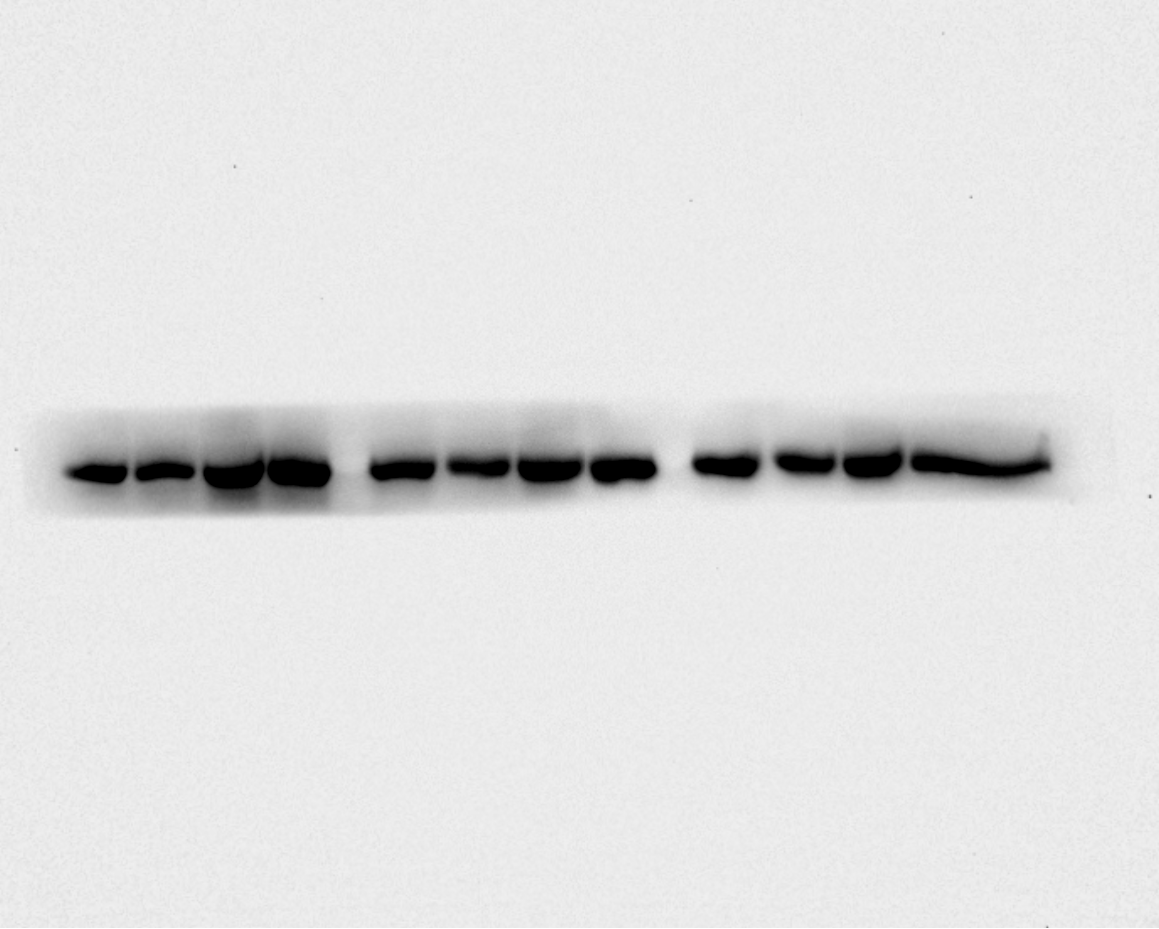

Supplement: Supplementary file 5 — Source data Fig. 1 [file 44321_2025_240_MOESM5_ESM.zip › Fig.1/1E_ImageData/chemidoc123 2020-10-30 13h03m13s(Chemiluminescence)_HSP90.tif]

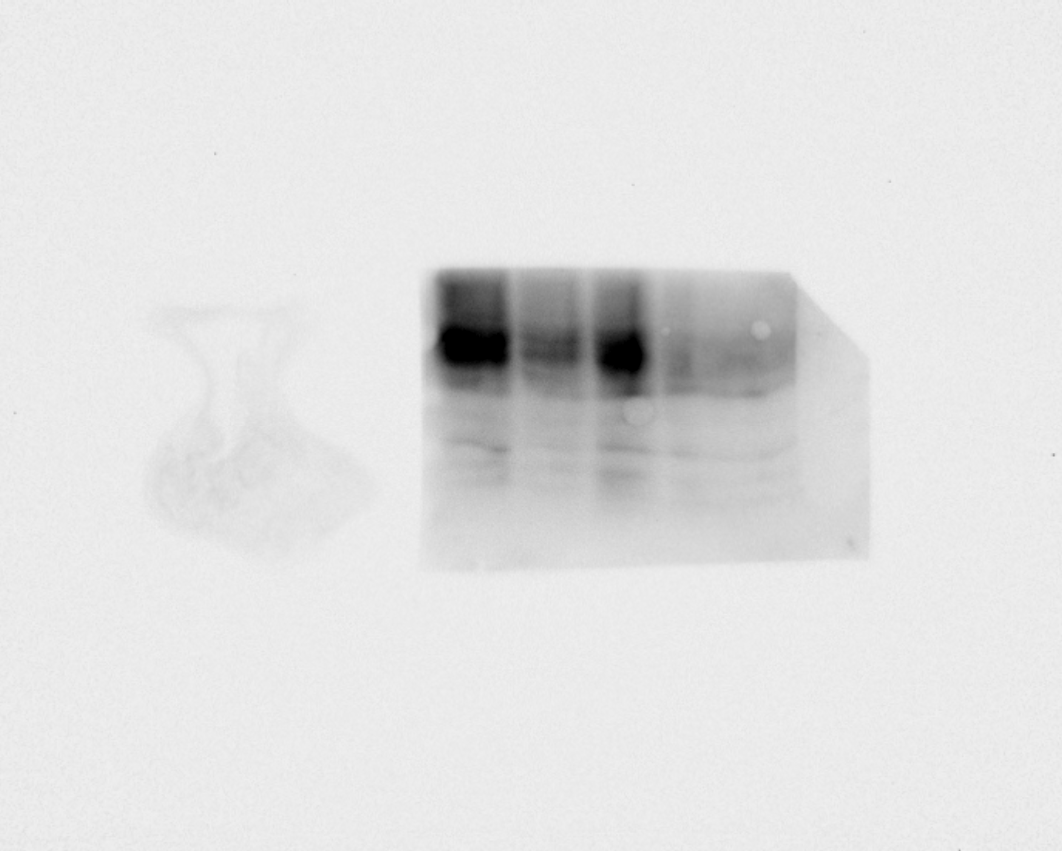

Supplement: Supplementary file 5 — Source data Fig. 1 [file 44321_2025_240_MOESM5_ESM.zip › Fig.1/1E_ImageData/chemidoc123 2020-10-30 13h27m14s(Chemiluminescence)_GLUT1.tif]

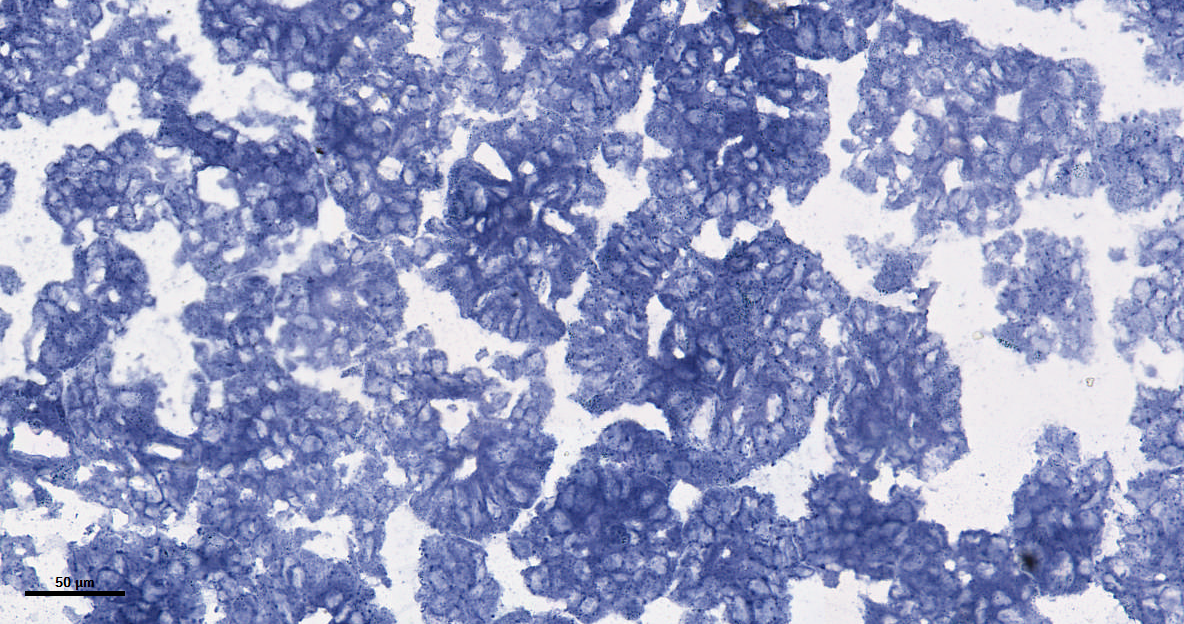

Supplement: Supplementary file 6 — Source data Fig. 2 [file 44321_2025_240_MOESM6_ESM.zip › Fig.2/2E_ImageData/G6PDH_AKP_20x.tif]

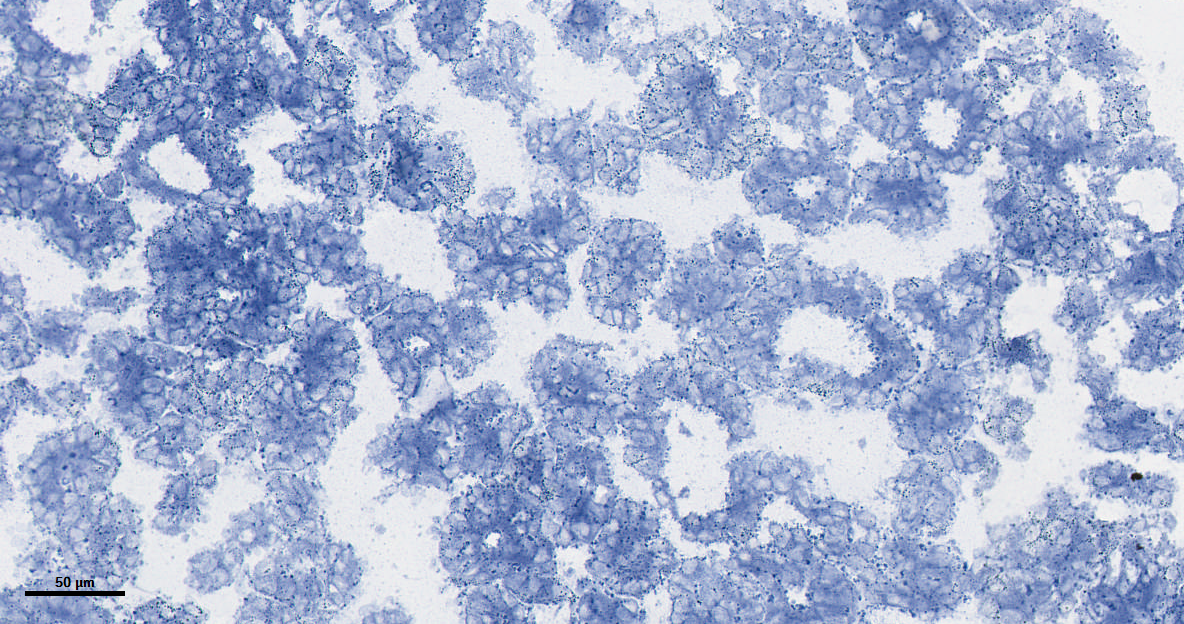

Supplement: Supplementary file 6 — Source data Fig. 2 [file 44321_2025_240_MOESM6_ESM.zip › Fig.2/2E_ImageData/G6PDH_AKPE_20x.tif]

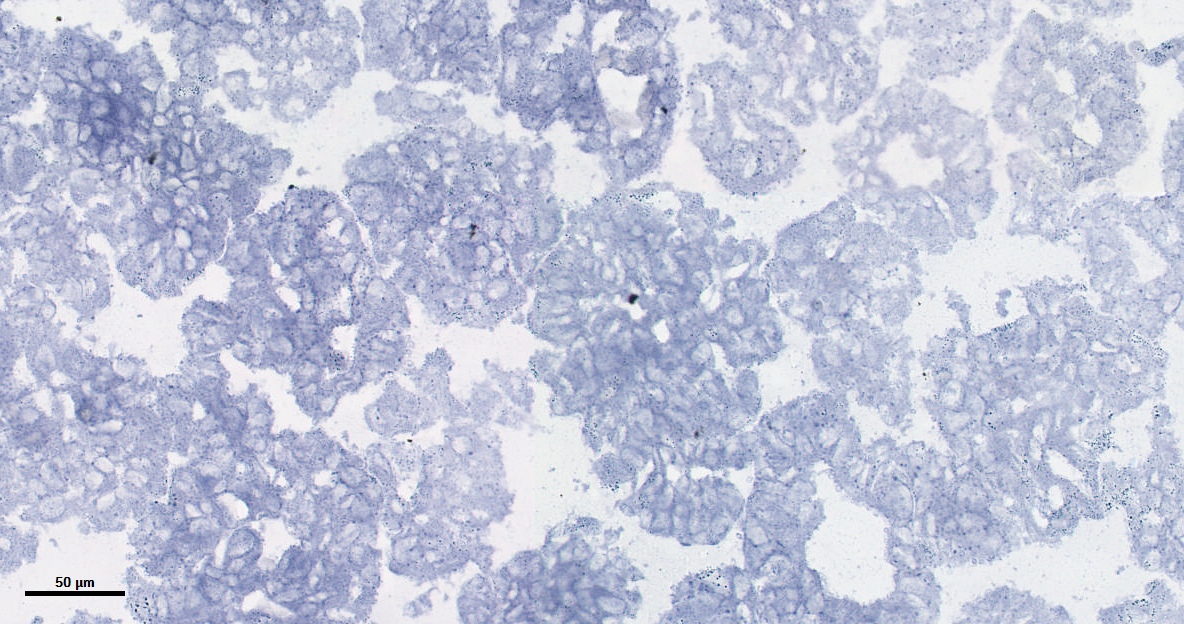

Supplement: Supplementary file 6 — Source data Fig. 2 [file 44321_2025_240_MOESM6_ESM.zip › Fig.2/2E_ImageData/GAPDH_AKP_20x.tif]

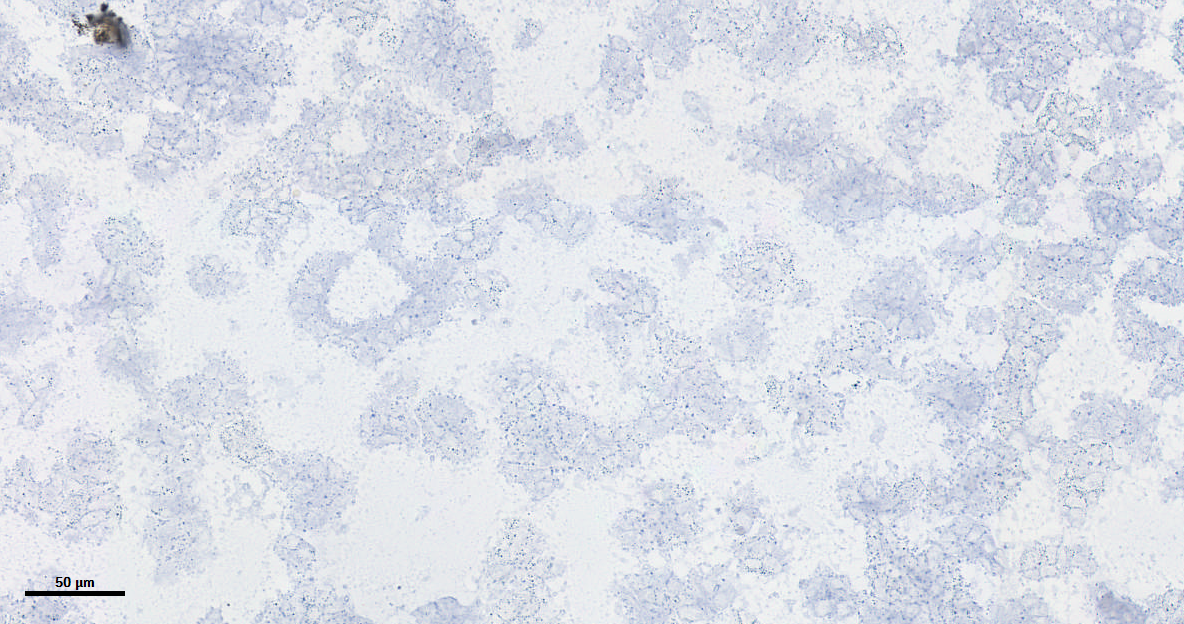

Supplement: Supplementary file 6 — Source data Fig. 2 [file 44321_2025_240_MOESM6_ESM.zip › Fig.2/2E_ImageData/GAPDH_AKPE_20x.tif]

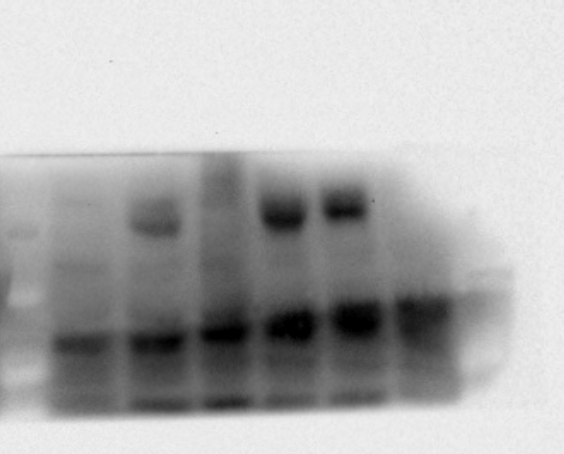

Supplement: Supplementary file 7 — Source data Fig. 4 [file 44321_2025_240_MOESM7_ESM.zip › Fig.4/4H_ImageData/dev-chemidoc 2023-11-23 15h11m57s(Chemiluminescence)_SMOC2.tif]

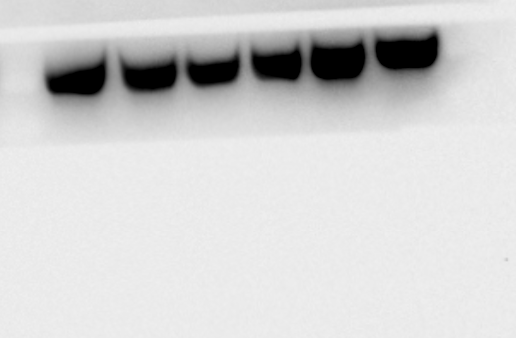

Supplement: Supplementary file 7 — Source data Fig. 4 [file 44321_2025_240_MOESM7_ESM.zip › Fig.4/4H_ImageData/dev-chemidoc 2023-11-23 15h07m31s(Chemiluminescence)_HSP90.tif]

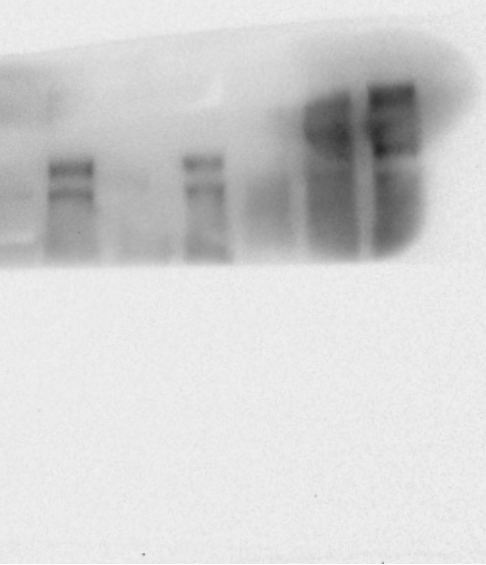

Supplement: Supplementary file 7 — Source data Fig. 4 [file 44321_2025_240_MOESM7_ESM.zip › Fig.4/4H_ImageData/dev-chemidoc 2023-11-23 15h05m42s(Chemiluminescence)_EGFR.tif]

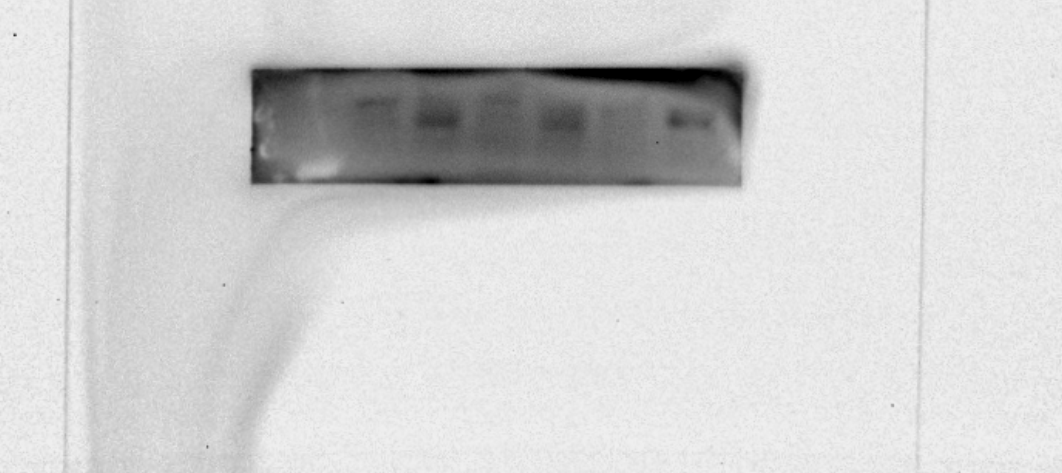

Supplement: Supplementary file 7 — Source data Fig. 4 [file 44321_2025_240_MOESM7_ESM.zip › Fig.4/4F_ImageData/chemidoc123 2023-03-14 11h11m59s(Chemiluminescence)_LEF1.tif]

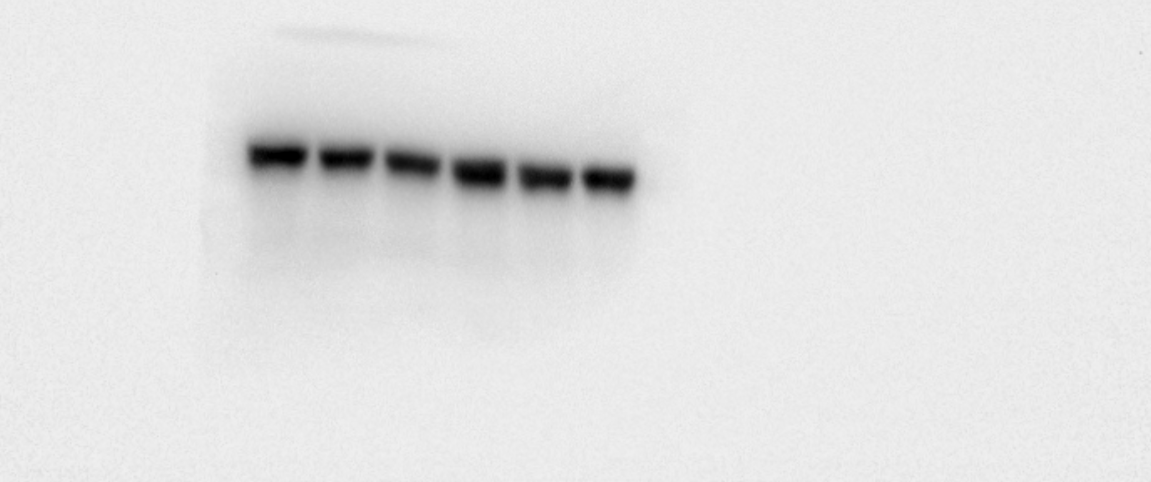

Supplement: Supplementary file 7 — Source data Fig. 4 [file 44321_2025_240_MOESM7_ESM.zip › Fig.4/4F_ImageData/chemidoc123 2023-03-14 10h54m45s(Chemiluminescence)_GSK3b.tif]

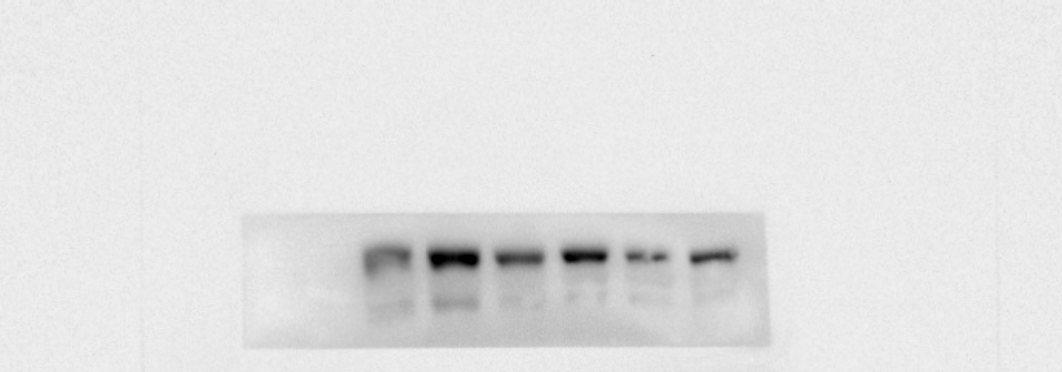

Supplement: Supplementary file 7 — Source data Fig. 4 [file 44321_2025_240_MOESM7_ESM.zip › Fig.4/4F_ImageData/chemidoc123 2023-03-14 10h58m09s(Chemiluminescence)_b-catenin.tif]

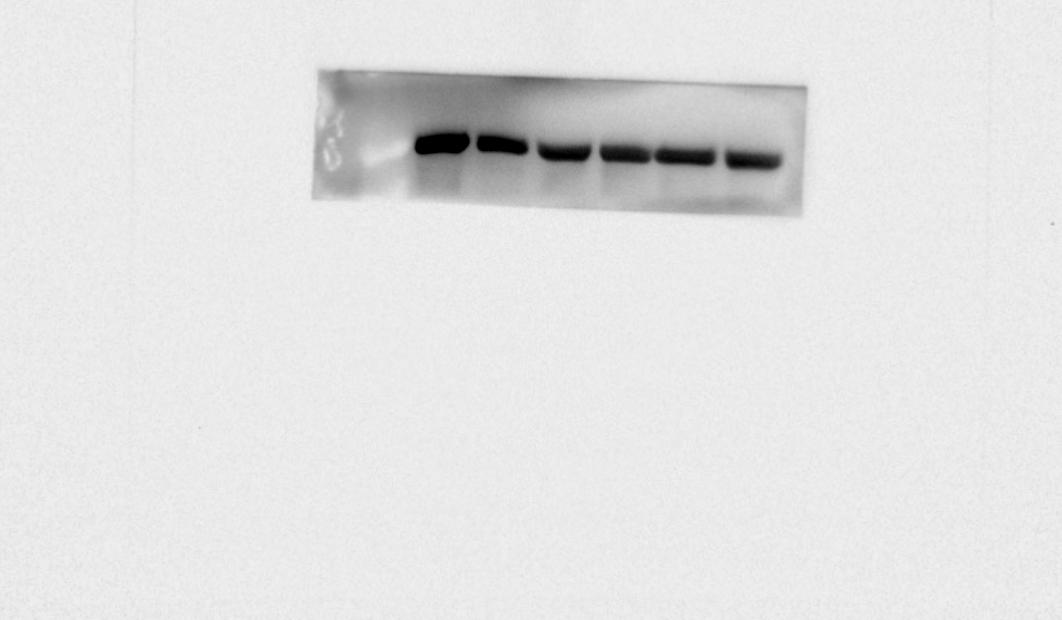

Supplement: Supplementary file 7 — Source data Fig. 4 [file 44321_2025_240_MOESM7_ESM.zip › Fig.4/4F_ImageData/chemidoc123 2023-03-13 11h24m49s(Chemiluminescence)_HSP90.tif]

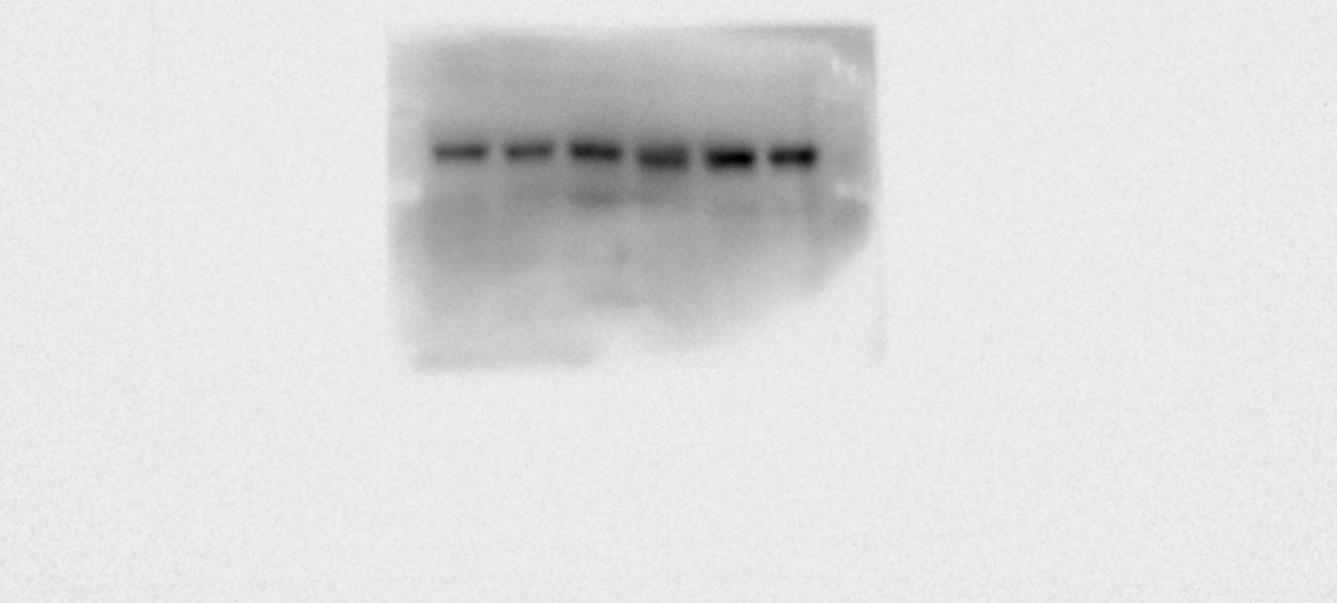

Supplement: Supplementary file 7 — Source data Fig. 4 [file 44321_2025_240_MOESM7_ESM.zip › Fig.4/4F_ImageData/chemidoc123 2023-03-13 11h34m10s(Chemiluminescence)_pGSK3b.tif]

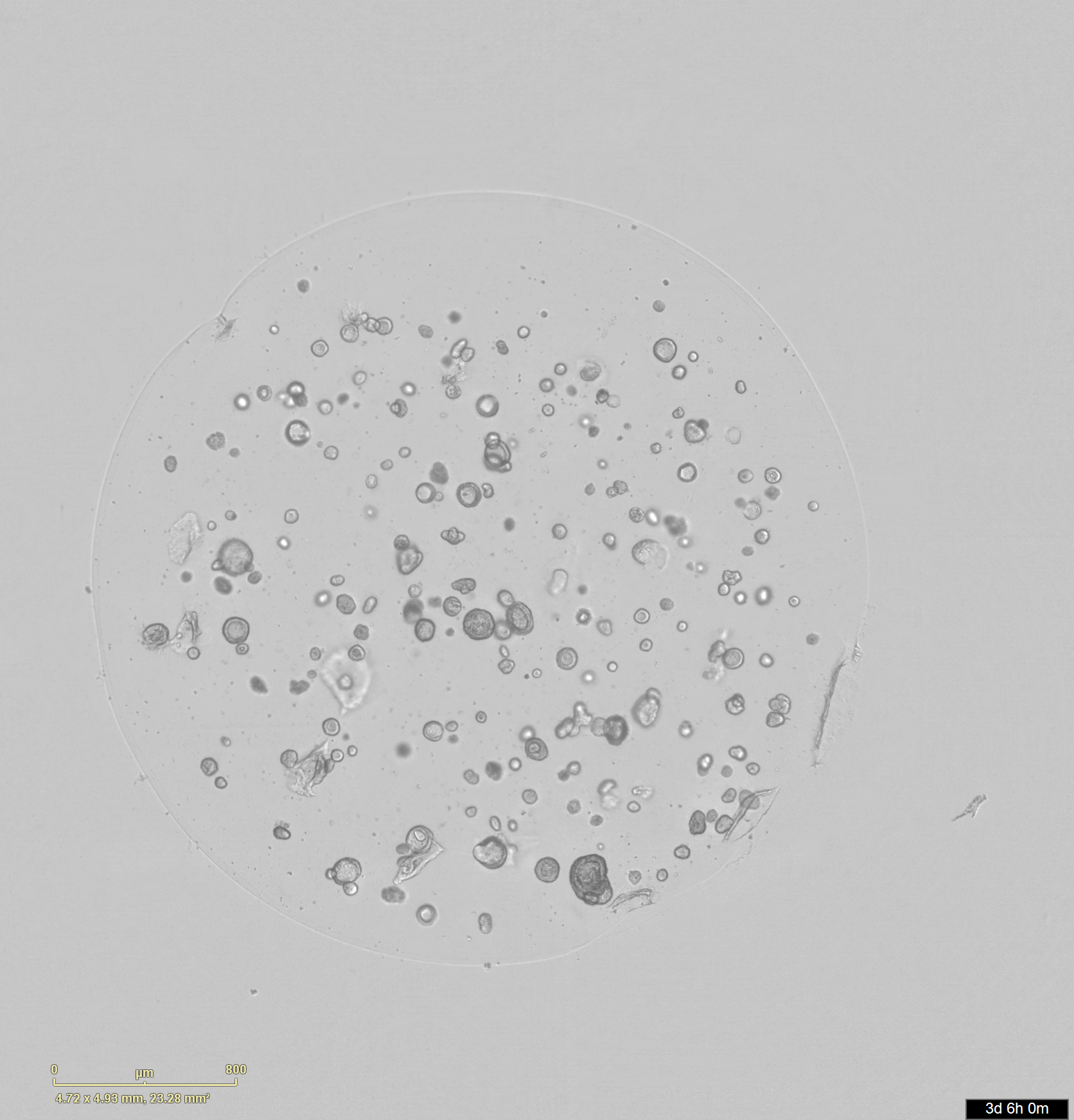

Supplement: Supplementary file 8 — Source data Fig. 5 [file 44321_2025_240_MOESM8_ESM.zip › Fig.5/5A_ImageData/AKPE_VID1057_D4_1_03d06h00m.tif]

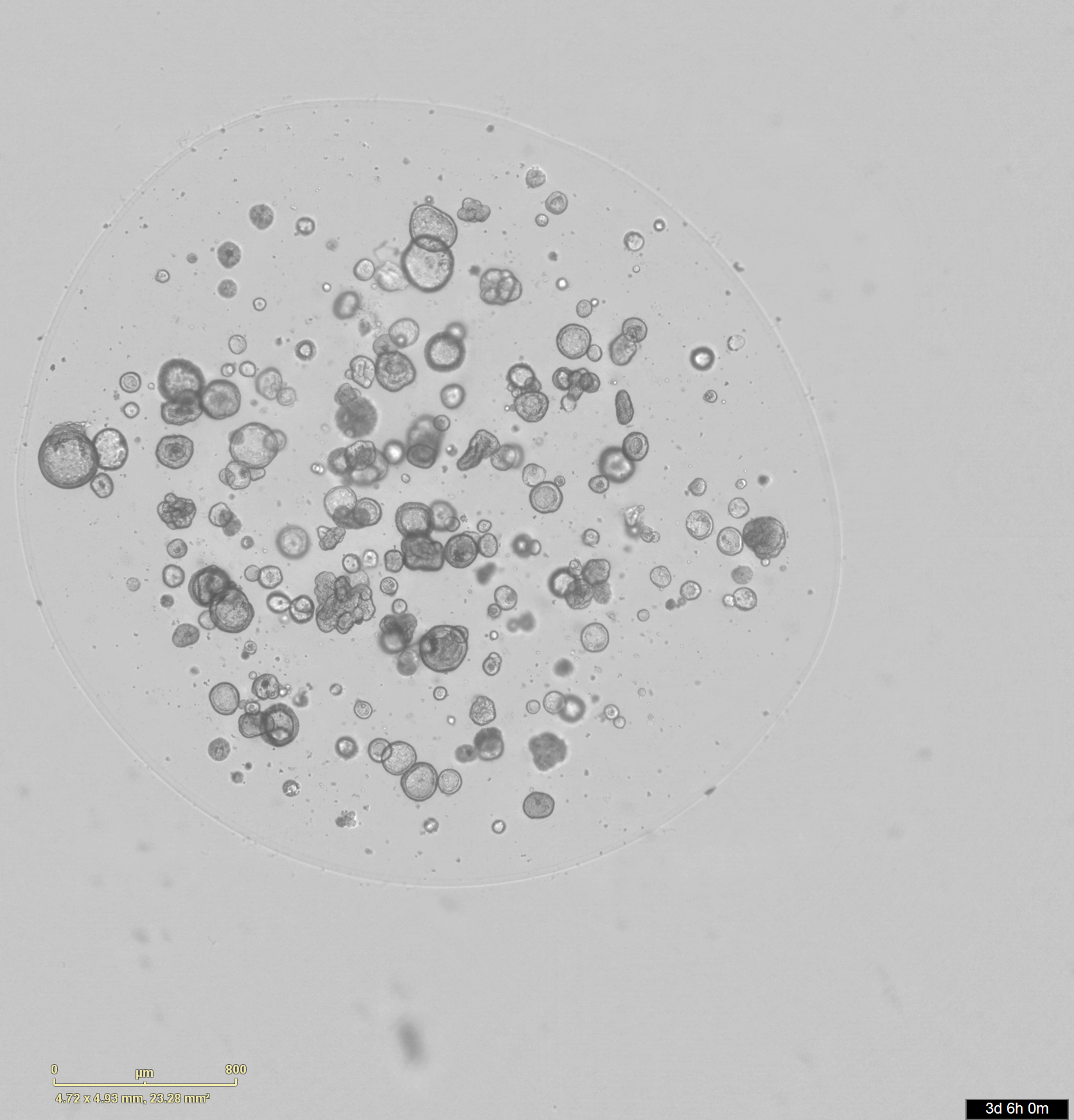

Supplement: Supplementary file 8 — Source data Fig. 5 [file 44321_2025_240_MOESM8_ESM.zip › Fig.5/5A_ImageData/AKP_VID1057_C3_1_03d06h00m.tif]
