# Supplementary material for: EGFR controls transcriptional and metabolic rewiring in KRASG12D colorectal cancer
Source: EMBO Mol Med. 2025 May 6;17(6):1355–92. doi: 10.1038/s44321-025-00240-4 (PMC12162862; doi:10.1038/s44321-025-00240-4)
Supplement: Supplementary file 10 — Expanded View Figures [file 44321_2025_240_MOESM10_ESM.pdf]

Expanded View Figures

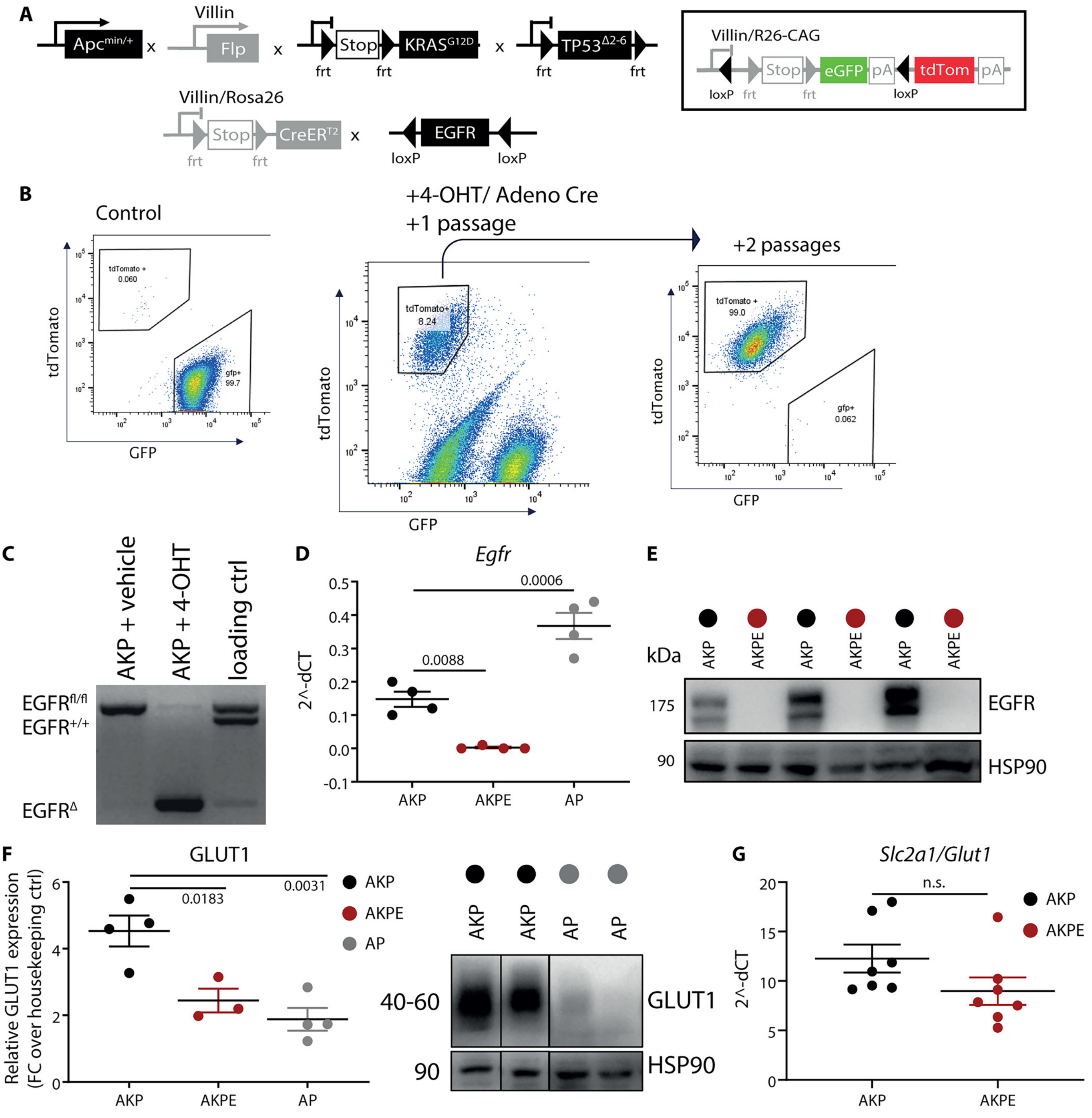

Figure EV1. Establishment and molecular characterization of EGFR-deficient organoids isolated from GEMMs.

(**A**) Schematic depiction of genetic crossing strategy used for generating GEMMs, from which organoids were derived. (**B**) Representative FACS gating strategy of AKP (green fluorescent protein (GFP)-positive), Tamoxifen (4-OHT) or Adeno-Cre induced recombination in AKP organoids after passage one (tdTomato and GFP positive) or sorted AKPE organoids (tdTomato positive), two passages after recombination (left). Depiction of genetic dual-reporter cassette (right). (**C**) PCR verification of genomic DNA from AKP plus vehicle, AKP plus Tamoxifen (4-OHT) or loading control (ctrl) samples for wild-type *Egfr*$^{+/+}$, floxed *Egfr*$^{fl/+}$ or recombined *Egfr*$^{\Delta}$ alleles. (**D**) RT-qPCR analysis of *Egfr* mRNA in AKP, AKPE or AP organoids; $n = 4$, One-way ANOVA. (**E**) Absence of EGFR protein in the three independently derived EGFR-deleted AKPE organoids used in this study. (**F**) Quantification (left) of absolute GLUT1 protein amount between AKP, AKPE, and AP organoids assessed by western blot analysis (right), One-way ANOVA ($n = 4$, 3 or 4, respectively). (**G**) RT-qPCR analysis of *Slc2a1/Glut1* mRNA expression in AKP or AKPE organoids ($n = 7$). All data represent mean +/− SEM. *P*-values calculated by paired, two-tailed t-test (between pairs of AKP and AKPE organoids) or One-way ANOVA.

© The Author(s)

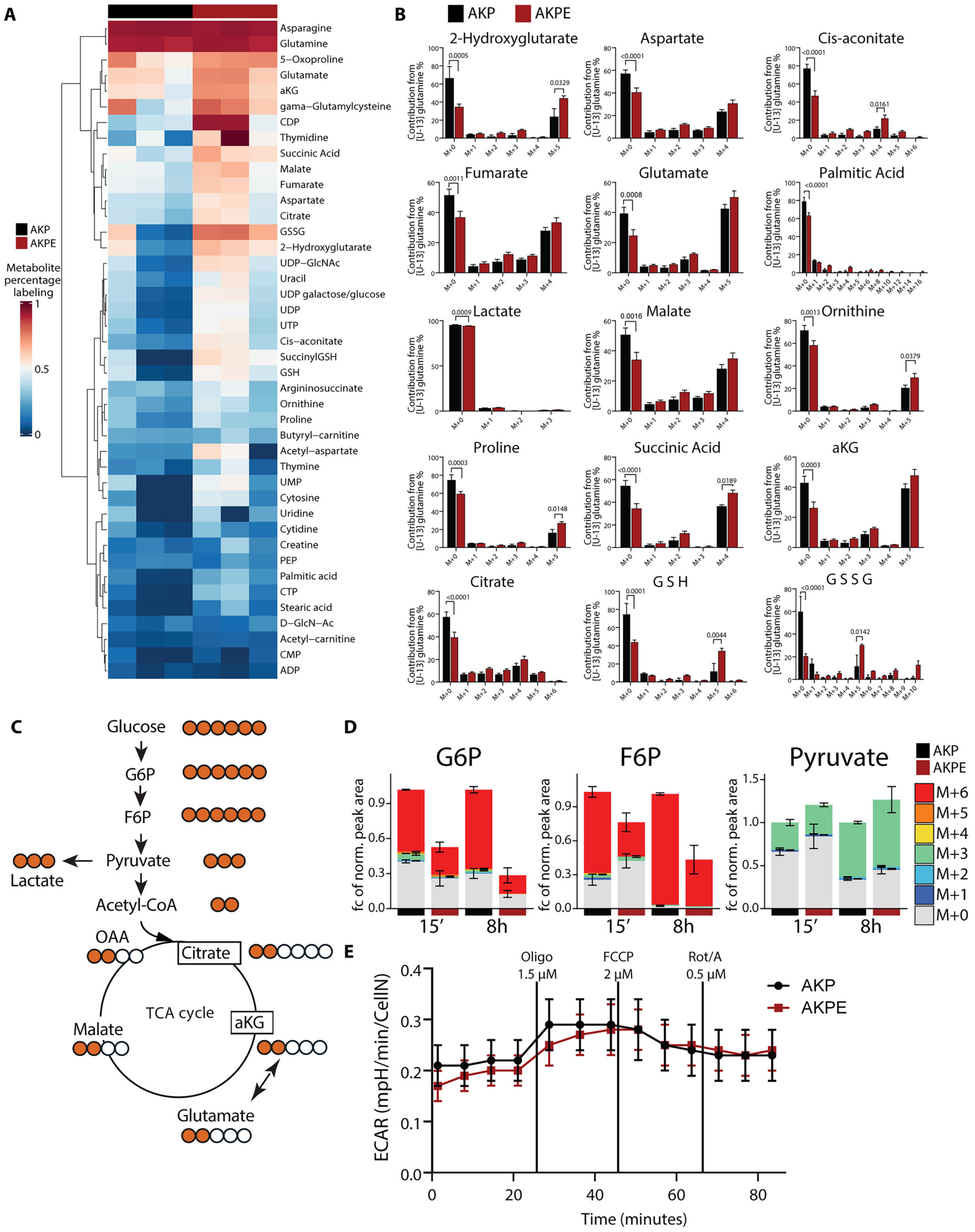
A
AKP
AKPE
Metabolite percentage labeling
Asparagine
Glutamine
5–Oxoproline
Glutamate
aKG
gama–Glutamylcysteine
CDP
Thymidine
Succinic Acid
Malate
Fumarate
Aspartate
Citrate
GSSG
2–Hydroxyglutarate
UDP–GlcNAc
Uracil
UDP galactose/glucose
UDP
UTP
Cis–aconitate
SuccinylGSH
GSH
Argininosuccinate
Ornithine
Proline
Butyryl–carnitine
Acetyl–aspartate
Thymine
UMP
Cytosine
Uridine
Cytidine
Creatine
PEP
Palmitic acid
CTP
Stearic acid
D–GlcN–Ac
Acetyl–carnitine
CMP
ADP
B
AKP AKPE
2-Hydroxyglutarate
Aspartate
Cis-aconitate
Fumarate
Glutamate
Palmitic Acid
Lactate
Malate
Ornithine
Proline
Succinic Acid
aKG
Citrate
GSH
GSSG
Contribution from [U-13] glutamine %
C
Glucose
G6P
F6P
Pyruvate
Lactate
Acetyl-CoA
OAA
Citrate
TCA cycle
aKG
Malate
Glutamate
D
G6P
F6P
Pyruvate
fc of norm. peak area
15′
8h
AKP
AKPE
M+6
M+5
M+4
M+3
M+2
M+1
M+0
E
Oligo 1.5 µM
FCCP 2 µM
Rot/A 0.5 µM
ECAR (mpH/min/CellN)
Time (minutes)
AKP
AKPE

Figure EV2. Stable isotope tracing and metabolic analysis reveals differential glutamine and glucose metabolism in EGFR-deficient organoids.

(**A**) Heatmap showing fractional labeling of respective metabolites in AKP or AKPE organoids. The color scale corresponds to the z-score value of relative abundance of the metabolite. (**B**) Fractional enrichment in ^{13}C-glutamine derived isotopologues of indicated metabolites as determined by LC-MS analysis. $M + 0$ (all carbons unlabeled) to $M + n$ isotopologues indicate number of ^{13}C atoms present in respective metabolite ($n = 3$ biological organoids with 4 technical replicates per organoid line).
(**C**) Schematic overview of glycolytic and oxidative catabolism and fractional contribution of labeled ^{13}C carbons of indicated metabolites. (**D**) Glucose tracer metabolomics in AKPE ($n = 2$, biological) or AKP ($n = 2$, biological) organoids (technical replicates 6 per organoid line) showing fold-change at timepoints 15 min and 8 h of fractional labeling of glucose-6-phosphate (G6P), fructose-6-phosphate (F6P), pyruvate. $M + 0$ (all carbons unlabeled) to $M + n$ isotopologues indicate number of ^{13}C atoms present in respective metabolite. Total abundance normalized to protein (BCA) content. (**E**) Representative extracellular acidification rate (ECAR) measurement of AKP and AKPE organoids ($n = 3$, biological) obtained by the Mito Stress test of the Seahorse XF analysis. Oligomycin, FCCP, Rotenone (Rot) and antimycin-A (A) were added at indicated timepoints. aKG: alpha-ketoglutarate. G6P: Glucose-6-phosphate. TCA: tricarboxylic acid. All data represent mean $+/-$ SEM. *P*-values calculated by paired, two-tailed t-test (between pairs of AKP and AKPE organoids).

 © The Author(s)

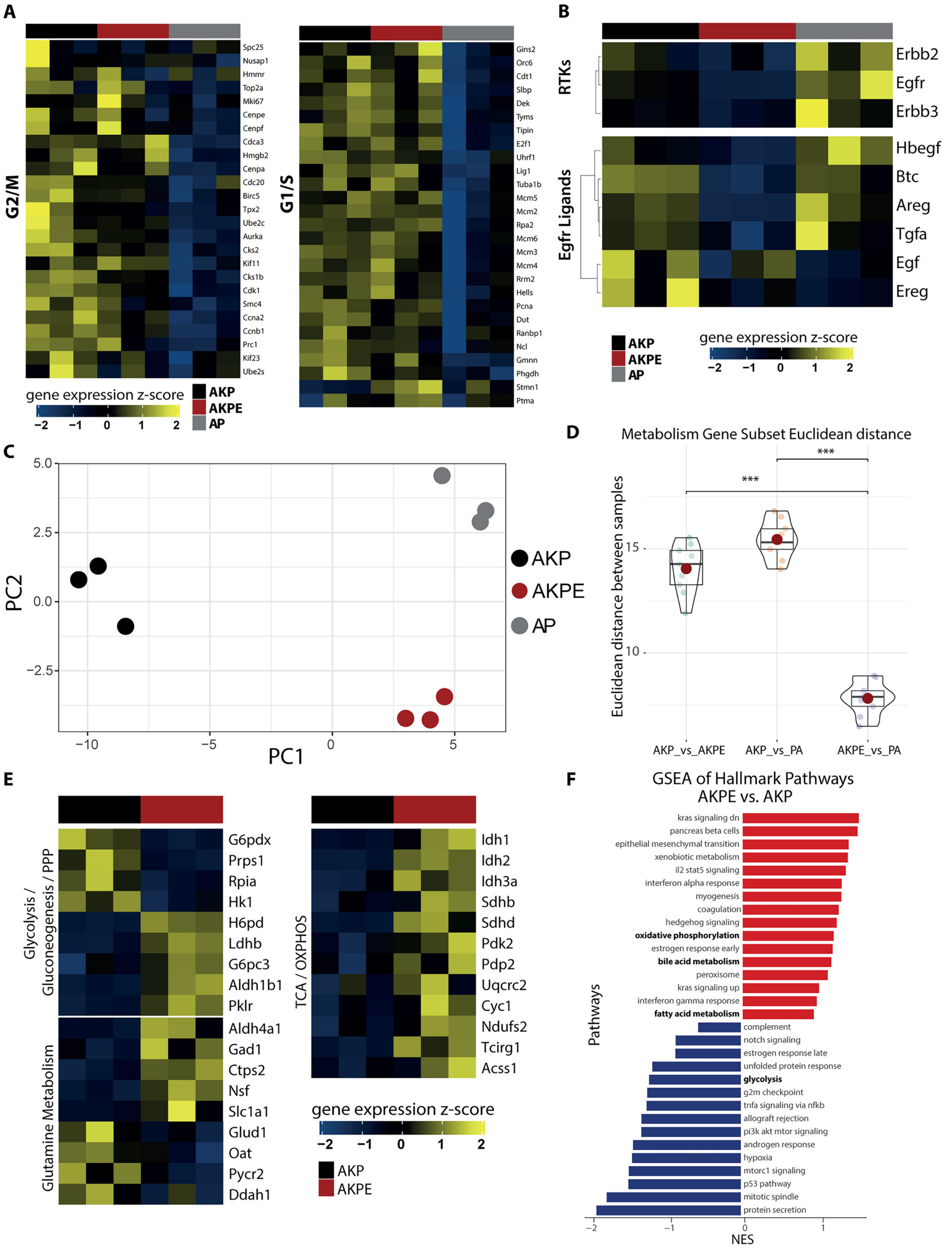
A
G2/M
Spc25
Nusap1
Hmmr
Top2a
Mki67
Cenpe
Cenpf
Cdca3
Hmgb2
Cenpa
Cdc20
Birc5
Tpx2
Ube2c
Aurka
Cks2
Kif11
Cks1b
Cdk1
Smc4
Ccna2
Ccnb1
Prc1
Kif23
Ube2s
gene expression z-score
−2 −1 0 1 2
AKP
AKPE
AP
G1/S
Gins2
Orc6
Cdt1
Slbp
Dek
Tyms
Tipin
E2f1
Uhrf1
Lig1
Tuba1b
Mcm5
Mcm2
Rpa2
Mcm6
Mcm3
Mcm4
Rrm2
Hells
Pcna
Dut
Ranbp1
Ncl
Gmnn
Phgdh
Stmn1
Ptma
B
RTKs
Erbb2
Egfr
Erbb3
Egfr Ligands
Hbegf
Btc
Areg
Tgfa
Egf
Ereg
AKP
AKPE
AP
gene expression z-score
−2 −1 0 1 2
C
PC2
PC1
5.0
2.5
0.0
−2.5
−10 −5 0 5
AKP
AKPE
AP
D
Metabolism Gene Subset Euclidean distance
Euclidean distance between samples
***
***
15
10
AKP_vs_AKPE
AKP_vs_PA
AKPE_vs_PA
E
Glycolysis / Gluconeogenesis / PPP
G6pdx
Prps1
Rpia
Hk1
H6pd
Ldhb
G6pc3
Aldh1b1
Pklr
Glutamine Metabolism
Aldh4a1
Gad1
Ctps2
Nsf
Slc1a1
Glud1
Oat
Pycr2
Ddah1
TCA / OXPHOS
Idh1
Idh2
Idh3a
Sdhb
Sdhd
Pdk2
Pdp2
Uqcrc2
Cyc1
Ndufs2
Tcirg1
Acss1
gene expression z-score
−2 −1 0 1 2
AKP
AKPE
F
GSEA of Hallmark Pathways
AKPE vs. AKP
Pathways
kras signaling dn
pancreas beta cells
epithelial mesenchymal transition
xenobiotic metabolism
il2 stat5 signaling
interferon alpha response
myogenesis
coagulation
hedgehog signaling
oxidative phosphorylation
estrogen response early
bile acid metabolism
peroxisome
kras signaling up
interferon gamma response
fatty acid metabolism
complement
notch signaling
estrogen response late
unfolded protein response
glycolysis
g2m checkpoint
tnfa signaling via nfkb
allograft rejection
pi3k akt mtor signaling
androgen response
hypoxia
mtorc1 signaling
p53 pathway
mitotic spindle
protein secretion
−2 −1 0 1
NES

Figure EV3. Distinct transcriptional changes induced by KRAS^{G12D} expression and EGFR deletion.

(**A**) Heatmap showing expression of selected cell cycle progression genes (based on (Avraham-Davidi et al, 2024)) of AKP, AKPE, and AP transcripts. (**B**) Heatmap showing expression of selected gene transcripts of receptor tyrosine kinases (RTKs) and EGFR ligands in AKPE, AKPE or AP organoids. (**C**) Principal component analysis (PCA) of metabolic gene subset of AKP, AKPE and AP organoids in steady state. Each subpopulation is depicted by indicated color. (**D**) Euclidean distance between samples of first and second principal component of metabolic gene subset between annotated comparisons, depicting comparisons of each sample to all other samples ($n = 9$). Horizontal lines denote the median and dots the mean. The box limits indicate 25th and 75th percentiles, whiskers extend to 1.5× of the interquartile range (IQR) from the 25th and 75th percentiles. (**E**) Heatmap showing expression of metabolic genes between AKP and AKPE organoids categorized according to molecular metabolic functional pathways. (**F**) Gene set enrichment analysis (GSEA) showing MSigDB Hallmark pathways significantly enriched in AKPE versus AKP ranked by their normalized enrichment score (NES). All data represent mean $+/-$ SEM. *P*-values calculated by One-way ANOVA.

 © The Author(s)

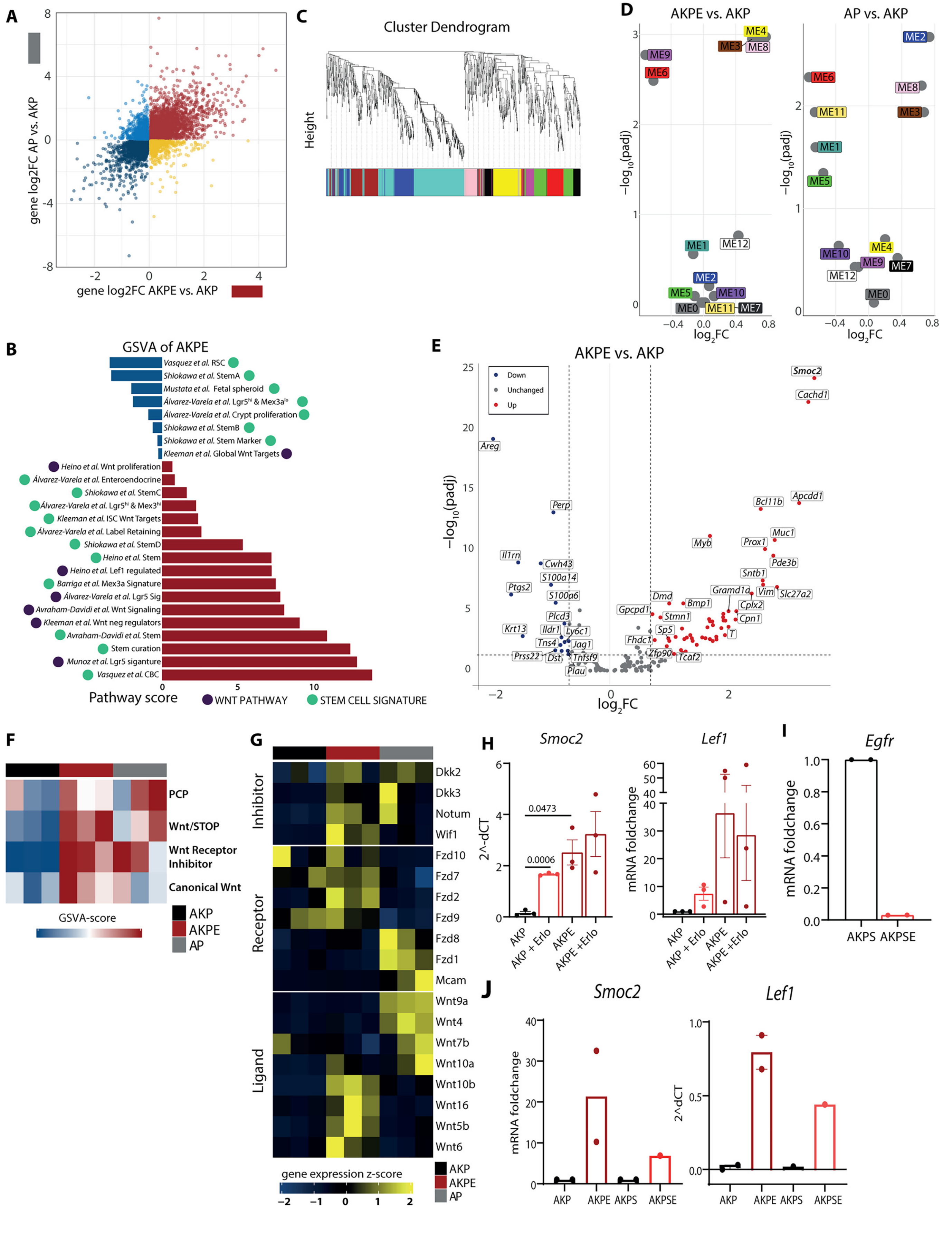
A
gene log2FC AP vs. AKP
gene log2FC AKPE vs. AKP
B
GSVA of AKPE
Vasquez et al. RSC
Shiokawa et al. StemA
Mustata et al. Fetal spheroid
Álvarez-Varela et al. Crypt proliferation
Shiokawa et al. StemB
Shiokawa et al. Stem Marker
Kleeman et al. Global Wnt Targets
Heino et al. Wnt proliferation
Álvarez-Varela et al. Enteroendocrine
Shiokawa et al. StemC
Kleeman et al. ISC Wnt Targets
Álvarez-Varela et al. Label Retaining
Shiokawa et al. StemD
Heino et al. Stem
Heino et al. Lef1 regulated
Barriga et al. Mex3a Signature
Álvarez-Varela et al. Lgr5 Sig
Avraham-Davidi et al. Wnt Signaling
Kleeman et al. Wnt neg regulators
Avraham-Davidi et al. Stem
Stem curation
Munoz et al. Lgr5 siganture
Vasquez et al. CBC
Pathway score
WNT PATHWAY
STEM CELL SIGNATURE
C
Cluster Dendrogram
Height
D
AKPE vs. AKP
AP vs. AKP
−log10(padj)
log2FC
ME0 ME1 ME2 ME3 ME4 ME5 ME6 ME7 ME8 ME9 ME10 ME11 ME12
E
AKPE vs. AKP
Down
Unchanged
Up
−log10(padj)
log2FC
Smoc2
Cachd1
Areg
Perp
Bcl11b
Apcdd1
Muc1
Myb
Prox1
Il1rn
Cwh43
Pde3b
S100a14
Sntb1
Ptgs2
S100a6
Gramd1a
Vim
Slc27a2
Dmd
Bmp1
Cplx2
Gpcpd1
Plcd3
Stmn1
Cpn1
Krt13
Ildr1
Ly6c1
Sp5
T
Fhdc1
Tns4
Jag1
Zfp90
Tcaf2
Prss22
Dst
Tnfsf9
Plau
F
PCP
Wnt/STOP
Wnt Receptor Inhibitor
Canonical Wnt
GSVA-score
AKP
AKPE
AP
G
Inhibitor
Receptor
Ligand
Dkk2
Dkk3
Notum
Wif1
Fzd10
Fzd7
Fzd2
Fzd9
Fzd8
Fzd1
Mcam
Wnt9a
Wnt4
Wnt7b
Wnt10a
Wnt10b
Wnt16
Wnt5b
Wnt6
gene expression z-score
AKP
AKPE
AP
H
Smoc2
Lef1
2^-dCT
mRNA foldchange
0.0473
0.0006
AKP
AKP + Erlo
AKPE
AKPE +Erlo
I
Egfr
mRNA foldchange
AKPS
AKPSE
J
Smoc2
Lef1
mRNA foldchange
2^dCT
AKP
AKPE
AKPS
AKPSE

Figure EV4. Transcriptomic analysis reveals WNT pathway genes affected in EGFR-deficient colorectal organoids.

(**A**) Scatter plot of fold changes from differential expression of AKPE versus AKP plotted against AP versus AKP derived fold changes. (**B**) Pathway enrichment of gene set variation analysis (GSVA) in AKPE organoids ranked by their pathway score. (**C**) Dendrogram of marker genes obtained by weighted gene correlation network analysis (WGCNA) according to modules. (**D**) Volcano plot of pathway-level modules derived from WGCNA analysis. (**E**) Volcano plot representing up or down-regulated gene expression of AKPE-associated genes according to fold-change ($n = 3$). (**F**) Heatmap of GSVA analysis of indicated WNT-pathways. (**G**) Heatmap of selected WNT-receptor and ligand interaction genes. (**H**) RT-qPCR analysis of *Smoc2* and *Lef1* mRNA expression in AKP, AKPE and erlotinib treated organoids ($n = 3$). (**I**, **J**) RT-qPCR analysis of *Egfr, Smoc2, Lef1* mRNA expression in AKPS or AKPSE organoids ($n = 2$). All data show mean +/− SEM, or for I data mean +/− SD. *P*-values calculated by paired, two-tailed t-test (between pairs of AKP and AKPE organoids).

 © The Author(s)

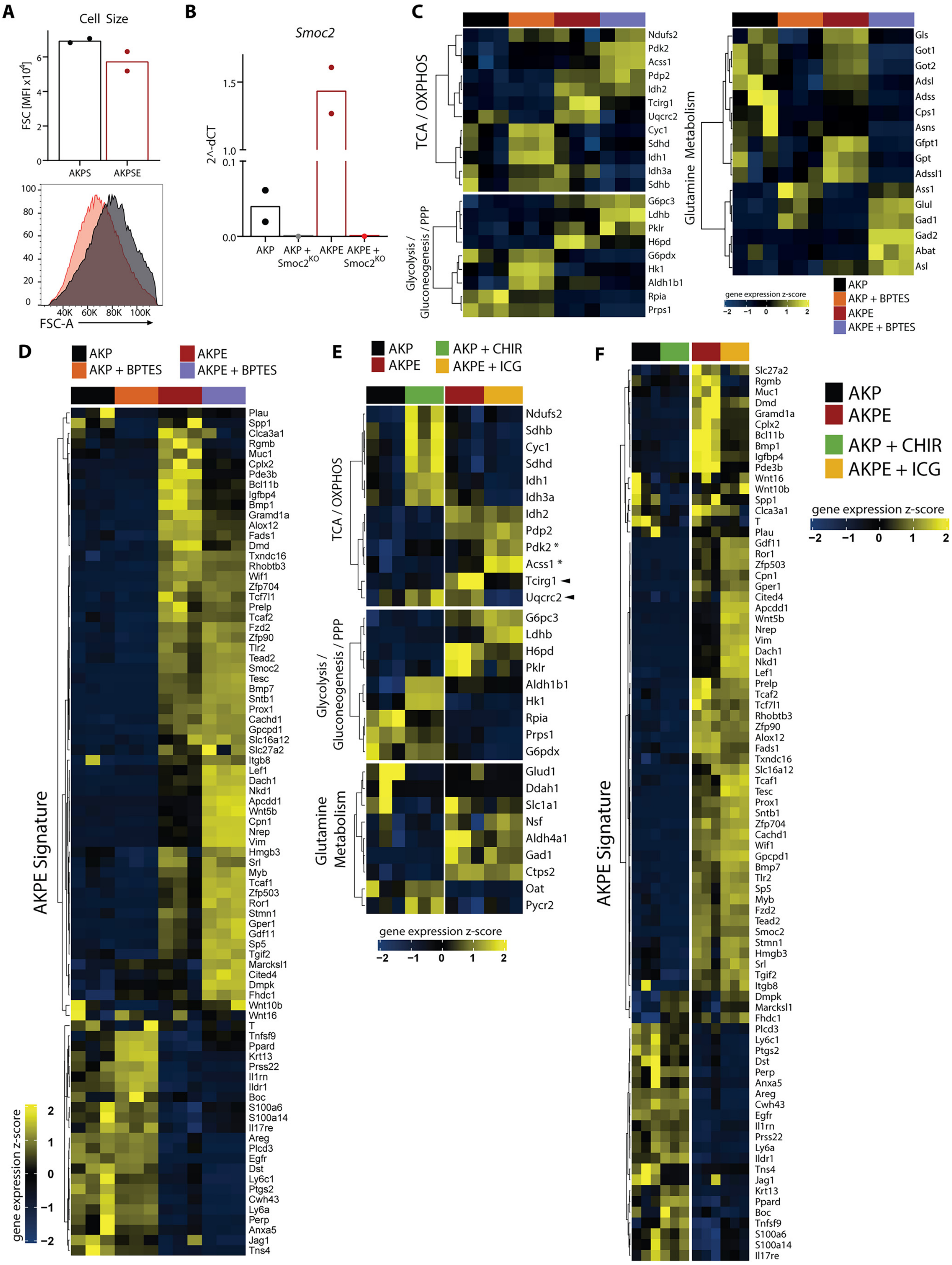
A
Cell Size
FSC [MFI x10^4]
0 2 4 6
AKPS AKPSE
100 80 60 40 20 0
40K 60K 80K 100K
FSC-A
B
Smoc2
2^-dCT
0.0 0.1 1.0 1.5
AKP AKP + Smoc2KO AKPE AKPE + Smoc2KO
C
TCA / OXPHOS
Ndufs2 Pdk2 Acss1 Pdp2 Idh2 Tcirg1 Uqcrc2 Cyc1 Sdhd Idh1 Idh3a Sdhb
Glycolysis / Gluconeogenesis / PPP
G6pc3 Ldhb Pklr H6pd G6pdx Hk1 Aldh1b1 Rpia Prps1
Glutamine Metabolism
Gls Got1 Got2 Adsl Adss Cps1 Asns Gfpt1 Gpt Adssl1 Ass1 Glul Gad1 Gad2 Abat Asl
gene expression z-score
−2 −1 0 1 2
AKP
AKP + BPTES
AKPE
AKPE + BPTES
D
AKP
AKP + BPTES
AKPE
AKPE + BPTES
AKPE Signature
Plau Spp1 Clca3a1 Rgmb Muc1 Cplx2 Pde3b Bcl11b Igfbp4 Bmp1 Gramd1a Alox12 Fads1 Dmd Txndc16 Rhobtb3 Wif1 Zfp704 Tcf7l1 Prelp Tcaf2 Fzd2 Zfp90 Tlr2 Tead2 Smoc2 Tesc Bmp7 Sntb1 Prox1 Cachd1 Gpcpd1 Slc16a12 Slc27a2 Itgb8 Lef1 Dach1 Nkd1 Apcdd1 Wnt5b Cpn1 Nrep Vim Hmgb3 Srl Myb Tcaf1 Zfp503 Ror1 Stmn1 Gper1 Gdf11 Sp5 Tgif2 Marcksl1 Cited4 Dmpk Fhdc1 Wnt10b Wnt16 T Tnfsf9 Ppard Krt13 Prss22 Il1rn Ildr1 Boc S100a6 S100a14 Il17re Areg Plcd3 Egfr Dst Ly6c1 Ptgs2 Cwh43 Ly6a Perp Anxa5 Jag1 Tns4
gene expression z-score
2 1 0 −1 −2
E
AKP
AKPE
AKP + CHIR
AKPE + ICG
TCA / OXPHOS
Ndufs2 Sdhb Cyc1 Sdhd Idh1 Idh3a Idh2 Pdp2 Pdk2 * Acss1 * Tcirg1 ◄ Uqcrc2 ◄
Glycolysis / Gluconeogenesis / PPP
G6pc3 Ldhb H6pd Pklr Aldh1b1 Hk1 Rpia Prps1 G6pdx
Glutamine Metabolism
Glud1 Ddah1 Slc1a1 Nsf Aldh4a1 Gad1 Ctps2 Oat Pycr2
gene expression z-score
−2 −1 0 1 2
F
AKPE Signature
Slc27a2 Rgmb Muc1 Dmd Gramd1a Cplx2 Bcl11b Bmp1 Igfbp4 Pde3b Wnt16 Wnt10b Spp1 Clca3a1 T Plau Gdf11 Ror1 Zfp503 Cpn1 Gper1 Cited4 Apcdd1 Wnt5b Nrep Vim Dach1 Nkd1 Lef1 Prelp Tcaf2 Tcf7l1 Rhobtb3 Zfp90 Alox12 Fads1 Txndc16 Slc16a12 Tcaf1 Tesc Prox1 Sntb1 Zfp704 Cachd1 Wif1 Gpcpd1 Bmp7 Tlr2 Sp5 Myb Fzd2 Tead2 Smoc2 Stmn1 Hmgb3 Srl Tgif2 Itgb8 Dmpk Marcksl1 Fhdc1 Plcd3 Ly6c1 Ptgs2 Dst Perp Anxa5 Areg Cwh43 Egfr Il1rn Prss22 Ly6a Ildr1 Tns4 Jag1 Krt13 Ppard Boc Tnfsf9 S100a6 S100a14 Il17re
AKP
AKPE
AKP + CHIR
AKPE + ICG
gene expression z-score
−2 −1 0 1 2

Figure EV5. Characterisation of organoids lacking Smoc2.

(**A**) Forward scatter mean fluorescent intensity (MFI) and representative histogram of AKPS and AKPSE organoids assessed by flow cytometry. Data show mean +/− SEM. (**B**) RT-qPCR analysis of *Smoc2* mRNA in AKP, AKP + Smoc2^{KO} or AKPE and AKPE + Smoc2^{KO} organoids ($n = 2$). (**C**) Heatmap showing subset of metabolic genes in AKP, AKPE or AKP and AKPE treated with control vehicle or 10 µM BPTES. (**D**) Heatmap showing expression of AKPE-signature genes in AKP, AKPE or AKP and AKPE treated with control vehicle or 10 µM BPTES. (**E**) Heatmap showing a subset of metabolic genes in AKP, AKPE, or AKP and AKPE treated with control vehicle or 3 µM CHIR99021 or 2 µM ICG-001. (**F**) Heatmap showing expression of AKPE-signature genes in AKP, AKPE, or AKP and AKPE treated with control vehicle or 3 µM CHIR99021 or 2 µM ICG-001.

 © The Author(s)

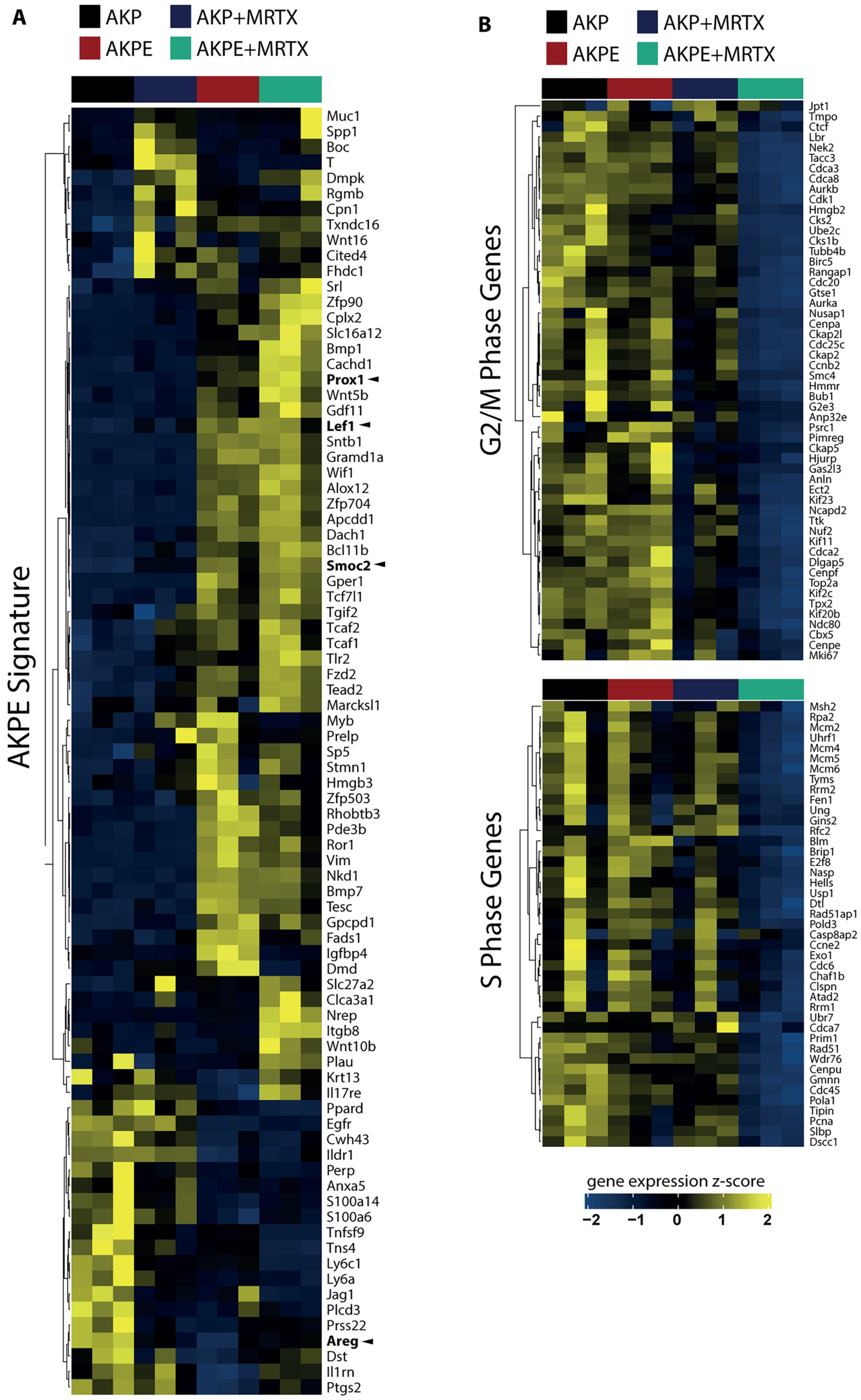
A
AKP
AKP+MRTX
AKPE
AKPE+MRTX
AKPE Signature
Muc1
Spp1
Boc
T
Dmpk
Rgmb
Cpn1
Txndc16
Wnt16
Cited4
Fhdc1
Srl
Zfp90
Cplx2
Slc16a12
Bmp1
Cachd1
Prox1 ◄
Wnt5b
Gdf11
Lef1 ◄
Sntb1
Gramd1a
Wif1
Alox12
Zfp704
Apcdd1
Dach1
Bcl11b
Smoc2 ◄
Gper1
Tcf7l1
Tgif2
Tcaf2
Tcaf1
Tlr2
Fzd2
Tead2
Marcksl1
Myb
Prelp
Sp5
Stmn1
Hmgb3
Zfp503
Rhobtb3
Pde3b
Ror1
Vim
Nkd1
Bmp7
Tesc
Gpcpd1
Fads1
Igfbp4
Dmd
Slc27a2
Clca3a1
Nrep
Itgb8
Wnt10b
Plau
Krt13
Il17re
Ppard
Egfr
Cwh43
Ildr1
Perp
Anxa5
S100a14
S100a6
Tnfsf9
Tns4
Ly6c1
Ly6a
Jag1
Plcd3
Prss22
Areg ◄
Dst
Il1rn
Ptgs2
B
AKP
AKP+MRTX
AKPE
AKPE+MRTX
G2/M Phase Genes
Jpt1
Tmpo
Ctcf
Lbr
Nek2
Tacc3
Cdca3
Cdca8
Aurkb
Cdk1
Hmgb2
Cks2
Ube2c
Cks1b
Tubb4b
Birc5
Rangap1
Cdc20
Gtse1
Aurka
Nusap1
Cenpa
Ckap2l
Cdc25c
Ckap2
Ccnb2
Smc4
Hmmr
Bub1
G2e3
Anp32e
Psrc1
Pimreg
Ckap5
Hjurp
Gas2l3
Anln
Ect2
Kif23
Ncapd2
Ttk
Nuf2
Kif11
Cdca2
Dlgap5
Cenpf
Top2a
Kif2c
Tpx2
Kif20b
Ndc80
Cbx5
Cenpe
Mki67
S Phase Genes
Msh2
Rpa2
Mcm2
Uhrf1
Mcm4
Mcm5
Mcm6
Tyms
Rrm2
Fen1
Ung
Gins2
Rfc2
Blm
Brip1
E2f8
Nasp
Hells
Usp1
Dtl
Rad51ap1
Pold3
Casp8ap2
Ccne2
Exo1
Cdc6
Chaf1b
Clspn
Atad2
Rrm1
Ubr7
Cdca7
Prim1
Rad51
Wdr76
Cenpu
Gmnn
Cdc45
Pola1
Tipin
Pcna
Slbp
Dscc1
gene expression z-score
−2
−1
0
1
2

© The Author(s)

Figure EV6. Distinct response to KRAS inhibition highlights EGFR loss as primary driver of AKPE gene signature.

(**A**) Heatmap showing expression of AKPE-signature genes in AKP, AKPE, or AKP and AKPE treated with control vehicle or MRTX1133 (100 nM). (**B**) Heatmap showing expression of selected cell cycle progression genes in AKP, AKPE, or AKP and AKPE treated with control vehicle or MRTX1133 (100 nM).

 © The Author(s)

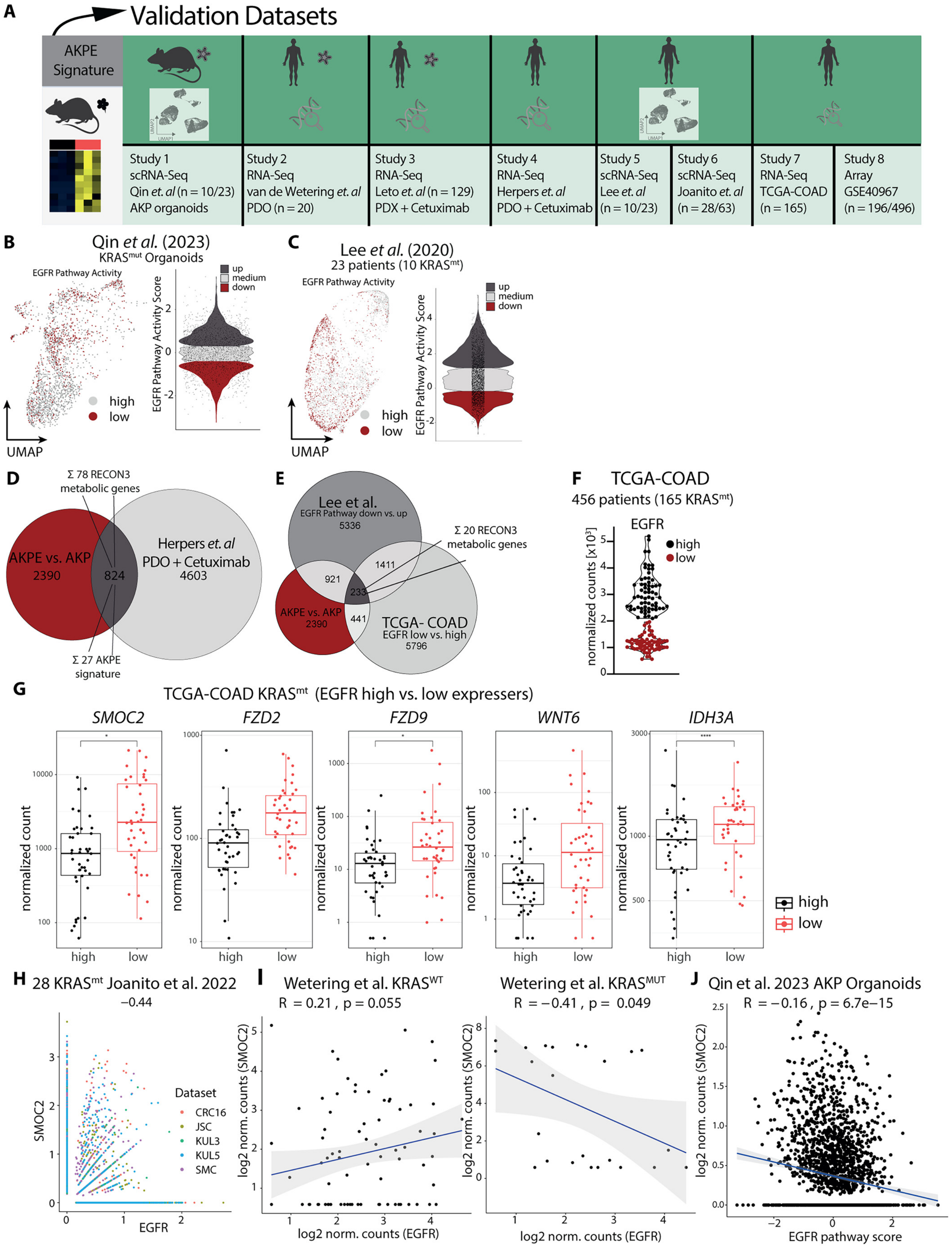
A
Validation Datasets
AKPE Signature
Study 1 scRNA-Seq Qin et. al (n = 10/23) AKP organoids
Study 2 RNA-Seq van de Wetering et. al PDO (n = 20)
Study 3 RNA-Seq Leto et. al (n = 129) PDX + Cetuximab
Study 4 RNA-Seq Herpers et. al PDO + Cetuximab
Study 5 scRNA-Seq Lee et. al (n = 10/23)
Study 6 scRNA-Seq Joanito et. al (n = 28/63)
Study 7 RNA-Seq TCGA-COAD (n = 165)
Study 8 Array GSE40967 (n = 196/496)
B
Qin et al. (2023)
KRASmut Organoids
EGFR Pathway Activity
high
low
UMAP
EGFR Pathway Activity Score
up
medium
down
C
Lee et al. (2020)
23 patients (10 KRASmt)
EGFR Pathway Activity
high
low
UMAP
EGFR Pathway Activity Score
up
medium
down
D
Σ 78 RECON3 metabolic genes
AKPE vs. AKP 2390
824
Herpers et. al PDO + Cetuximab 4603
Σ 27 AKPE signature
E
Lee et al. EGFR Pathway down vs. up 5336
Σ 20 RECON3 metabolic genes
921
1411
233
AKPE vs. AKP 2390
441
TCGA- COAD EGFR low vs. high 5796
F
TCGA-COAD
456 patients (165 KRASmt)
EGFR
high
low
normalized counts [x10³]
G
TCGA-COAD KRASmt (EGFR high vs. low expressers)
SMOC2
FZD2
FZD9
WNT6
IDH3A
normalized count
high
low
H
28 KRASmt Joanito et al. 2022
−0.44
SMOC2
EGFR
Dataset
CRC16
JSC
KUL3
KUL5
SMC
I
Wetering et al. KRASWT
R = 0.21 , p = 0.055
Wetering et al. KRASMUT
R = −0.41 , p = 0.049
log2 norm. counts (SMOC2)
log2 norm. counts (EGFR)
J
Qin et al. 2023 AKP Organoids
R = −0.16 , p = 6.7e−15
log2 norm. counts (SMOC2)
EGFR pathway score

Figure EV7. Validation of EGFR-associated AKPE signatures across CRC datasets from mouse organoids and human tumors.

(**A**) Graphical overview of accessed, publicly available mouse and human CRC datasets. *n* indicate number of $KRAS^{mt}$ of all samples. (**B**) Classification of single cells based on EGFR high or low pathways activity from Qin et al, 2023 ($n = 10$ organoids). (**C**) Classification of single cells based on EGFR high or low pathways activity from Lee et al, 2020 ($n = 10$ patients). (**D**) Venn diagram of mutually expressed genes between AKPE versus AKP, EGFR high versus low expressors from Herpers et al PDOs treated with cetuximab or control vehicle. (**E**) Stratification of TCGA-COAD $KRAS^{mt}$ patients into high and low *EGFR* expressors ($n = 165$ patients). (**F**) Venn diagram of mutually expressed genes between AKPE versus AKP, *EGFR* high vs. low expressors from TCGA-COAD and cells with down versus upregulated EGFR pathway signature from Lee et al, 2020. (**G**) Normalized counts of *SMOC2*, *FZD2*, *FZD9*, *WNT6* and *IDH3A* of TCGA-COAD $KRAS^{mt}$ patients of *EGFR* high and low expressors ($n = 165$ patients). Horizontal lines denote the median, box limits indicate 25th and 75th percentiles and whiskers extend from the hinge to the lowest/largest value no further than 1.5x IQR from the 25th and 75th percentiles. (**H**) Scatter correlation plots of *EGFR* versus *SMOC2* expression in $KRAS^{mt}$ single cells of the Joanito et al dataset. (**I**) Correlation of *EGFR* versus *SMOC2* expression in $KRAS^{wt}$ or mt patients of the Wetering et al dataset. (**J**) Correlation of *EGFR* versus *SMOC2* in single cells of AKP organoids in the Qin et al dataset.

 © The Author(s)
